# A Multimodal Auxiliary Classification System for Osteosarcoma Histopathological Images Based on Deep Active Learning

**DOI:** 10.3390/healthcare10112189

**Published:** 2022-10-31

**Authors:** Fangfang Gou, Jun Liu, Jun Zhu, Jia Wu

**Affiliations:** 1 School of Computer Science and Engineering, Central South University, Changsha 410083, China; 2 The Second People’s Hospital of Huaihua, Huaihua 418000, China; 3 The First People’s Hospital of Huaihua, Huaihua 418000, China; 4 Collaborative Innovation Center for Medical Artificial Intelligence and Big Data Decision Making Assistance, Hunan University of Medicine, Huaihua 418000, China; 5 Research Center for Artificial Intelligence, Monash University, Melbourne, VIC 3800, Australia

**Keywords:** medical system, active learning, auxiliary diagnosis of osteosarcoma, histopathological images, classification, 68T01

## Abstract

Histopathological examination is an important criterion in the clinical diagnosis of osteosarcoma. With the improvement of hardware technology and computing power, pathological image analysis systems based on artificial intelligence have been widely used. However, classifying numerous intricate pathology images by hand is a tiresome task for pathologists. The lack of labeling data makes the system costly and difficult to build. This study constructs a classification assistance system (OHIcsA) based on active learning (AL) and a generative adversarial network (GAN). The system initially uses a small, labeled training set to train the classifier. Then, the most informative samples from the unlabeled images are selected for expert annotation. To retrain the network, the final chosen images are added to the initial labeled dataset. Experiments on real datasets show that our proposed method achieves high classification performance with an AUC value of 0.995 and an accuracy value of 0.989 using a small amount of labeled data. It reduces the cost of building a medical system. Clinical diagnosis can be aided by the system’s findings, which can also increase the effectiveness and verifiable accuracy of doctors.

## 1. Introduction

The incidence of osteosarcoma ranks first among malignant bone tumors [1]. As a highly malignant tumor, it is extremely harmful to human health. Adolescents and the elderly are the most affected groups. Although the incidence of osteosarcoma is only 3 to 10 per million, it accounts for 40% of primary malignant bone tumors [2]. Osteosarcoma has high mortality and morbidity rates, especially in developing countries [3]. Due to the large population, the death toll is high. Early screening and diagnosis of osteosarcoma are critical to improving patient survival [4].

Routine detection methods for osteosarcoma include symptomatic examination, imaging examination, and pathological examination [5]. Since many tumors do not have typical imaging features, it is difficult to determine the nature of the mass by relying solely on imaging examinations, and it is impossible to determine whether a patient has a bone tumor [6]. Osteosarcoma has multiple subtypes with different pathological features [7]. Therefore, the pathological examination is regarded by all experts as the “gold standard” in the diagnosis of osteosarcoma.

Digital pathology images are obtained by scanning pathology slides. The data volume of pathological images is very large, and one histopathological section contains millions of cells [8]. The complex pathological features of osteosarcoma require extremely high professionalism and the experience of pathologists [9]. The number of specialized pathologists in a hospital is limited, and each specialist processes many slices in a day [10]. This is a very time-consuming and complicated job. In addition, the huge workload also makes doctors overworked [11]. Diagnosis results are subject to subjective influence, which can easily lead to missed diagnosis and misdiagnosis. Therefore, it is extremely important to develop a decision analysis system for histopathological images to assist pathologists in diagnosing osteosarcoma and alleviate the problems existing in hospitals.

With the development and popularization of artificial intelligence technology in the medical field, the neural network plays an increasingly important role in the field of medical image analysis with its powerful feature extraction ability [12,13,14], such as auxiliary staging of lung cancer, MRI segmentation of osteosarcoma [8], and other applications. However, many methods based on supervised learning still face the following problems when analyzing histopathological images of osteosarcoma:

(1) The system construction lacks enough labeled data, and the initial training set is insufficient [15]. Insufficient labeled data will greatly limit the performance of intelligent diagnostic systems based on supervised learning.
(2) Lots of unlabeled pathology images. The data volume of histopathological images in hospital databases is very large. However, labeled data are scarce.
(3) The cost of labeling samples is high. Due to the complexity of histopathological sections of bone and flesh, non-professionals cannot distinguish them. Only relying on professional pathologists to mark samples is costly and difficult.

To solve the problem that there are few pathological images of osteosarcoma labeled during model training, and data are difficult to obtain, active learning selects informative samples from unlabeled data and sends them to pathologists for labeling [16,17]. Such datasets are then trained to maximize model performance within a limited labeling budget. The methods of minimum confidence, marginal sampling, entropy, etc., are all based on the uncertainty of the model for the sample [18]. Such methods are biased and complex, which can affect the representativeness of the sample. Even though these active-learning techniques lessen the need for labeled training data, there are still some issues with relying solely on one tactic. The uncertainty-based metric alone is overconfident in sample selection, and the sample’s prior information is likely to be disregarded, resulting in unstable model performance. It may even be worse than the random sampling strategy. The selection of more samples with less information could result from only taking sample diversity into account, which would raise the cost of labeling.

Based on this, this study proposes an active-learning-based intelligent assisted classification system for osteosarcoma histopathological images (OHIcsA). The system effectively improves the annotation gain of osteosarcoma pathological images by actively acquiring the most characteristic pathological images as annotated samples. First, we train the network to reconstruct more pseudo-samples using the labeled dataset. The more insightful pathological images are then chosen from the unlabeled samples to be labeled using a selection strategy. These labeled images are then put together to retrain the network. Through this iterative training, selection, annotation, and retraining method, the OHIcsA method effectively alleviates the problems of high cost and difficulty in labeling osteosarcoma pathological images. In the clinical management of osteosarcoma, the results are used as a diagnostic aid, which can increase the precision and effectiveness of diagnosis and decrease the incidence of missed and misdiagnosis cases.

The main contributions of this research include the following:

(1) This paper builds an osteosarcoma classification system based on active learning and histopathology images. The system utilizes the fitting ability of deep neural networks to achieve image classification. It not only improves the efficiency of pathologists but also contributes to the objective accuracy of diagnosis.
(2) We combine generative adversarial networks (GAN) and active learning to obtain more high-value samples; GAN constructs high-quality pseudo-samples, and active learning obtains more unlabeled images with the “richest” information. This approach maximizes the performance of the model with a small number of labeled images. It effectively reduces the cost of labeling images, which is valuable in areas where there are not enough pathologists.
(3) A new strategy that integrates the query sample class diversity and uncertainty is proposed. It effectively reduces the problems of sampling bias that often result from uncertainty-based sampling alone and the increased tagging costs that can result from considering diversity alone. This approach effectively improves the ability of the model to select samples.
(4) Experimental results with osteosarcoma pathology images show that our system can achieve good classification performance with only a small number of labeled images. The results processed by the system can be used as an objective reference for clinical diagnosis and improve the detection accuracy of physicians.

## 2. Related Works

With the advancement of deep-learning technology and the improvement of big data computing capabilities, there are now many medical assistant decision-making methods. We introduce some methods.

The effectiveness of deep-learning methods in disease identification and detection was first demonstrated [19,20]. He et al. [21] proposed a multi-view uncertainty measurement method. By performing a softmax operation on each middle layer, the final uncertainty measurement takes into account the parameters of the middle layer of the model. Sunpreet Kaur Nanda et al. [22] developed an intelligent medical system that can quickly and accurately detect whether people are wearing a mask or not by using YOLO and CNN models. It lays the foundation for developing intelligent medical image systems for the detection of various disease and tumor abnormalities. Shivani G. Dharmale et al. [23] used machine-learning techniques to identify faults and anomalies in medical sensors from the perspective of medical sensors. They are mainly implemented through the random forest, k-neighborhood algorithm, and analysis based on geographical and temporal information. Neha Yadav et al. [24] proposed a hemorrhoid detection method (LeakyReLU) based on the HSV model. It includes deep-learning techniques such as K-Means, texture analysis, an HSV-based segmentation model, and activation function with Leaky ReLU. The method achieves a high detection accuracy.

When the task lacks sufficient samples, data augmentation can be performed by generating the model. Commonly used generative models include GAN and VAE. Many outstanding generative models can generate samples that conform to the original distribution without introducing excessive noise, improving the robustness of the model without reducing its performance of the model. Generative models are widely used in data augmentation [25]. They are also closely integrated with active learning. When lacking unlabeled samples, Zhu et al. [26] used GAN to generate samples. These samples are selected based on a specific strategy and handed to experts for labeling. In [27], Tran et al. combine the reconstructed samples of the generated model with the selected samples and train the ACGAN model and classifier. In both data augmentation [28] and active learning, samples need to be selected. The main difference is that the former is selected to be similar to the original sample, because the generative model may produce a lot of data that do not conform to the original data distribution, while the latter is to try to select the sample that is not similar to the original sample to reduce epistemic uncertainty.

K George et al. [29] utilized a deep-learned kernel feature classifier to detect breast cancer from histopathology images. V Gupta et al. [30] implemented an adenocarcinoma histopathology image classification based on the ResNet model. P Wang et al. [31] proposed a pathological image classification method (Cd-dtffNet). These methods have a very good role in promoting the diagnosis of breast cancer. Subsequently, to more accurately predict the size of tumors in breast cancer, Ophir Nave et al. [32] combined mathematical models and machine-learning algorithms. The study shows that employing mathematical models to discover image features can result in more precise forecasts.

Similarly, Sekhar et al. [33] used transfer learning to classify brain tumors in MRI images into three categories (glioma, meningioma, and pituitary). Wu et al. [34] designed an algorithm for segmenting lung nodules in chest CT images. The algorithm uses a 3D conditional random field (CRF) to continuously optimize training samples during training. The optimized samples accelerate the convergence of the 3D-UNet model and reduce the training time. Gur et al. [35] proposed a novel unsupervised method for vessel segmentation in pathological images. Based on edgeless morphological active contours, the method can label datasets and be applied to similar datasets.

Many researchers use computer vision technology to realize the auxiliary diagnosis of osteosarcoma. Long ago, Brian D Ragland et al. [36] performed a detailed analysis of osteosarcoma from a cytogenetic and biomolecular perspective, demonstrating that no cytogenetic and molecular markers have been identified to determine the diagnosis of osteosarcoma. After that, Rashika Mishra et al. [37] divided tumors into three groups based on CNN: living tumors, necrotic tumors, and non-tumors, and they also came up with a method to determine the amount of necrosis in pathological images. Anisuzzaman et al. [38] used transfer learning to supervise the metastases of bone tumors in bone and soft tissue. Yedong Shen et al. [39] proposed an OSGABN method based on guided aggregate bilateral networks. It mainly segmented MRI images through a fast bilateral segmentation network (FaBiNet), and the results showed that the method has high accuracy. Yu Fu [40] used a somatic neural network for the automatic classification of histological images of osteosarcoma, helping pathologists to improve the accuracy of diagnosis. To further improve the classification accuracy, Barzekar et al. [41] and D’Acunto et al. [42] used a machine-learning approach to differentiate between malignant and benign tumor cells.

## 3. System Design

**Figure 1:**
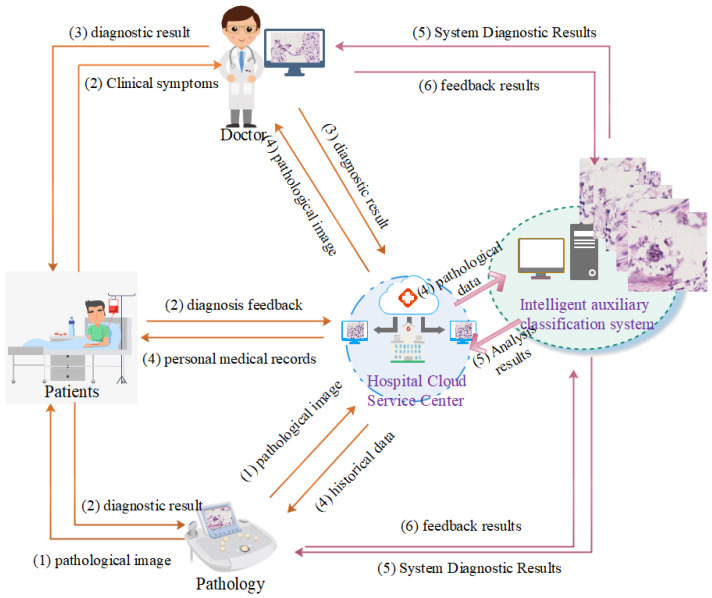
Patient pathology data decision-making process. The orange arrow is the workflow of the original medical system. The purple arrow is the workflow after adding the intelligent classification assistance system.

With the rapid development of large-scale data-computing capabilities and the continuous improvement of hardware technology, the research and application scope of artificial intelligence in the medical field continues to expand [43,44,45]. Especially in the field of medical images, AI uses its computer vision technology to realize lesion identification and labeling, image segmentation, feature extraction, comparative analysis, 3D reconstruction, etc. [46,47,48,49]. Due to the complexity of histopathological images of osteosarcoma, it is difficult for non-professionals to distinguish them [50,51]. The development of an automated and accurate histopathology image analysis system is of great importance to assist pathologists and clinical specialists in their work. As shown in Figure 1, the processing flow of pathological data of patients with osteosarcoma includes several parts: patient, pathology, doctor, hospital cloud service center, and intelligent auxiliary classification system. The specific process includes: (1) The pathology department sends pathology images to the patient and uploads them to the cloud service center. (2) The clinical diagnostic features of the patient are sent to the doctor and fed back to the cloud service center for storage. (3) The doctor gives the diagnosis to the patient and uploads it to the cloud service center. (4) Patients, pathology departments, physicians, and intelligent classification assistance systems receive data from the cloud service center continuously. (5) The results of the system analysis are provided to the physicians and pathology departments as an auxiliary reference for diagnosis and uploaded to the cloud service center. (6) The pathology department and the doctor will feed the results of the diagnosis to the intelligence system again.

However, in the diagnosis and treatment of osteosarcoma, the only way to label pathological data is to rely on professional pathologists. Therefore, labeling data is expensive and difficult. Although the application of active learning effectively alleviates the problem of requiring a large number of labeled samples for supervised learning model training, existing methods based only on uncertainty are overconfident and often result in sampling bias and data redundancy. Considering only diversity in turn leads to an increase in the cost of labeling, after considering the above issues together, this study proposes an osteosarcoma auxiliary diagnosis system based on active learning and generative adversarial networks (OHIcsA). It mainly improves the classification performance through the powerful fitting ability of the deep neural network. We combine active learning and generative adversarial networks. Active learning integrates the diversity and uncertainty of samples to obtain more informative samples. The adversarial model uses its powerful data generation capabilities to extend the original training set. This approach drives the model to obtain faster performance gains and maximizes the performance of the model using a small number of high-quality labeled samples.

The common symbols and their meanings in this section are shown in Table 1.

### 3.1. Models Design

**Figure 2:**
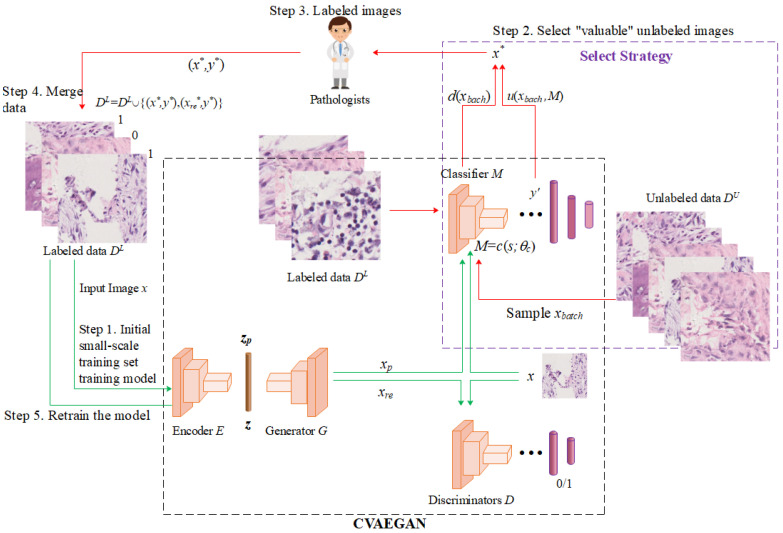
The detailed structure of the proposed deep active-learning framework. The boxes in the figure represent models, and the ellipses represent database entities. Oracle is usually a human expert. The arrows represent the data flow, the green arrows are the training process, and the red arrows are the filtering process. E, G, D, and M form the CVAEGAN model.

In this section and the following section, we will describe how the proposed framework works in detail. The patterns in histopathological images are complex and require large amounts of labeled data to train the model [52,53,54]. In order not to increase the annotation burden, we constructed a new classification model that combines active learning and generative adversarial models, choosing a conditional variational self-encoder (CVAEGAN) as the main framework. CVAEGAN combines generating adversarial networks (GAN) and variable score auto-encoder (VAE). It combines the ability of active learning to acquire the most informative samples and the ability of GAN to learn data distribution to generate real pseudo-samples simultaneously. We achieved faster model performance improvements than when employing only the uncertainty-based approach by feeding both selected and generated samples into the fundamental model for training. We used a convolutional neural network as a model for the classification task. Osteosarcoma pathology images and their classes are denoted by *x* and *y*, respectively. By using the data generation capability of the GAN model to extend the original training set, the system obtains faster performance improvements. This means that we can obtain higher performance with the same labeling cost and the same training method. As shown in Figure 2, in this regard, the structure of each module is as follows:

Auto-encoder: It is divided into two parts: encoder E and generator G. E is in charge of encoding and mapping the sample x to a potential vector representation z. Then, z=E(x), and the generator G is responsible for using E or reconstructing the original image based on the encoding result of z, to generate a new image $x_{re}$. Meanwhile, $z_{p}$ is randomly sampled from a specific distribution (we use here a normal distribution with mean 0 and variance 1, denoted as N (0,1)) and reconstructed by the generator G to generate the pseudo-image $x_{p}$. Thus, we consider that the learned low-dimensional variable z obeys the same distribution over the labeled and unlabeled samples. The auto-encoder is trained using all labeled data as well as unlabeled data. In turn, the model is guaranteed to process all samples fairly and without bias.

Discriminator D: It measures the difference in distribution between labeled data $D^{L}$ and unlabeled data $D^{U}$ and determines whether the image is a real image or a generated image. That is, the discriminator D is used to distinguish x from $x_{p}$ and $x_{re}$. The large of $D^{U}$ is much larger than that of $D^{L}$ in the osteosarcoma pathology image.

Classifier M: In histopathological image classification, M classifies the labeled images to determine their classes. The parameters of the classifier are denoted by *θ*. M can be any model, which greatly increases the applicability of the framework.

Figure 2 in this paper shows the proposed deep active-learning model. Its main process consists of four steps:

(1) Step 1: an initial small-scale labeled training set $D^{L}$ is used to train the neural network model, including encoder E, generator G, discriminator D, and classifier M.
(2) Step 2: the most informative sample is selected from the large number of unlabeled samples.

The selection strategy will take into account both the class diversity of the samples and the uncertainty of the model on the samples. Moreover, it uses generative adversarial networks to reconstruct the selected samples to increase the diversity of the data. A subset of $x_{batch}$ is sampled according to a specific strategy. The strategy will be described in Section 3.2.

(3) Step 3: Pathology expert labeled data. After the pathology sample is labeled by the expert, it is put into the training set along with the reconstructed sample ($x_{re}$ in Figure 2) for the next iteration of training.
(4) Step 4 and Step 5: the selected sample data are merged into the original labeled dataset to retrain the classifier.

(1) $$D^{L}=D^{L}\cup \left\{\left(x^{*},y^{*}\right),\left(x_{re}^{*},y^{*}\right)\right\}$$

where $D^{L}$ is the labeled data, $\left(x^{*},y^{*}\right)$ is the pathological sample labeled by the pathologist, and $\left(x_{re}^{*},y^{*}\right)$ is the real sample generated by automatically coding it.

With this iterative training, selection, annotation, and retraining approach, our proposed framework achieves similar performance compared to training with a full labeled dataset but using only about half of the labeled data. This significantly reduces the cost of building such a medical system.

Under the above description, the mathematical model describing the classification of osteosarcoma pathology images is a system based on the CVAEGAN model. In the model, our goal is to minimize the following loss functions to obtain superior classification results:

(2) $$L_{CVAEGAN}=L_{C}+L_{D}+\lambda_{1}L_{KL}+\lambda_{2}L_{G}+\lambda_{3}L_{GD}+\lambda_{4}L_{GC}$$

where $\lambda_{1}$,$\;\lambda_{2}$,$\;\lambda_{3}$,$\;\lambda_{4}$ are hyperparameters to trade off different losses. $L_{C}$, $L_{D}$, $L_{KL}$, $L_{G}$, $L_{GD}$, $L_{GC}$ are losses for different optimization targets. The detailed expressions are given in Equations (3)–(8).

(3) $$L_{D}=-(log(D\left(x\right)+log(1-D\left(x_{re}\right)+log\left(1-D\left(x_{p}\right)\right)$$

$L_{D}$ is the loss of discriminator *D*. It aims to let *D* distinguish the true samples x from reconstructed samples $x_{re}$ and generated pseudo-samples $x_{p}$.

(4) $$L_{C}=-log(P(M(y|x))$$

$L_{C}$ is the loss of classifier M. It aims to force classifier M to output the true label y when giving a true sample x. In addition, the network tries to minimize the Softmax loss:

(5) $$L_{KL}=KL(q(z|x,y)||N\left(0,1\right))$$

$L_{KL}$ denotes the difference between distribution N(0,1) and posterior probability distribution of z when given x, y denoted by q(z|x,y). KL(⋅) is the Kullback–Leibler divergence operator. This will help z, the output of encoder E obeys a normal distribution, and the sampled pseudo-latent vector $z_{p}$ comes from the same sample distribution with true samples. In other words, it can help to make $z_{p}$ more real, thus contributing to generating a realistic pseudo-image $x_{p}$, where $x_{p}=G\left(z_{p}\right)$.

(6) $$L_{G}=\frac{1}{2}({\left||x-x_{re}|\right|}_{2}^{2}+{\left||f_{D}\left(x\right)-f_{D}\left(x_{re}\right)|\right|}_{2}^{2}+{\left||f_{M}\left(x\right)-f_{M}\left(x_{re}\right)|\right|}_{2}^{2})$$

$L_{G}$ denotes a part of the loss of generator G. It consists of three parts. The first one is the loss between the true sample (x) and the reconstructed sample ($x_{re}$). $x_{re}=G\left(E\left(x\right)\right)$. This part forces the encoder and generator coordinate to reconstruct the image more precisely. In the second part and third part, $f_{M}\left(\cdot \right)$ and $f_{D}\left(\cdot \right)$ mean the second-to-last layer’s output. The latter two parts evaluate the difference of the original sample (x) and the reconstructed sample ($x_{re}$) in the view of discriminator D and classifier M.

(7) $$L_{GD}=\frac{1}{2}{\left||\frac{1}{m}\sum_{i}^{m}f_{D}(x)-\frac{1}{m}\sum_{i}^{m}f_{D}\left(x_{p}\right)|\right|}_{2}^{2}$$

(8) $$L_{GC}=\frac{1}{2}{\left||\frac{1}{m}\sum_{c}f_{M}^{c_{i}}(x)-\frac{1}{m}\sum_{c}f_{M}^{c_{i}}\left(x_{p}\right)|\right|}_{2}^{2}$$

$L_{GD}$,$\;L_{GC}$ are also losses of generator G. Different from $L_{G}$, the force generator generates pseudo-samples $x_{p}$ that have close class centers to true samples x in the view of discriminator D and classifier M. The latter two items in $L_{G}$ focus on the singular sample’s difference between true sample x and pseudo-sample $x_{re}$, while $L_{GD}$,$\;L_{GC}$ focus on the difference among batch samples.

Through the collaborative training of multiple models, it is ensured that the generator G generates high-quality samples and classifier M also performs well. The main reason for choosing a conditional variational self-encoder (CVAEGAN) as the model in this paper is that it generates realistic images with a strong fit to the data distribution of the original samples and it can reconstruct x and generate $x_{re}$. $x_{re}$ is similar to but different from the original image x. It can be regarded as the original image with added noise. Adding it to the training set can increase the number of the training set and help train a classifier that is robust to noise.

### 3.2. Selection Strategy

**Figure 3:**
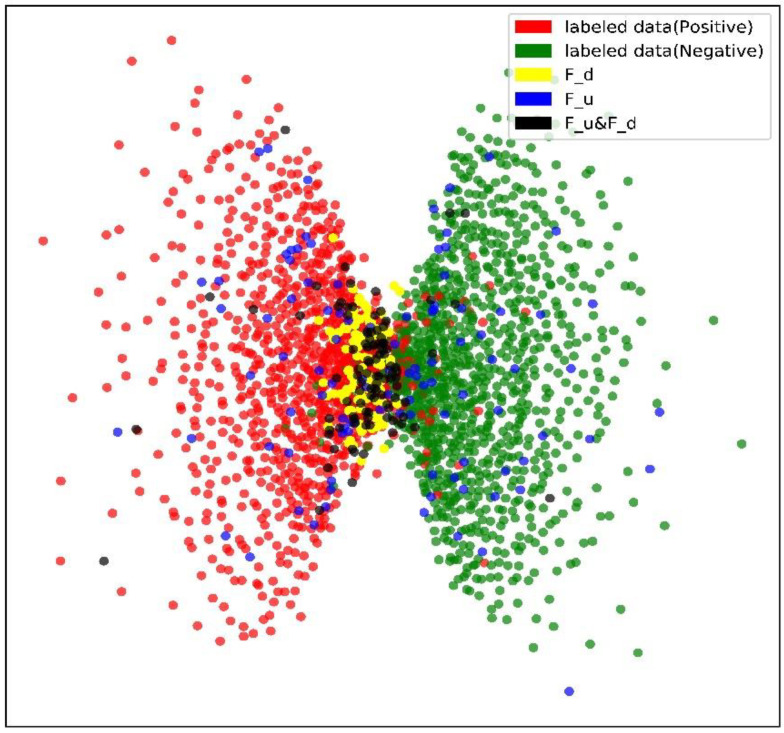
MDS visualization of three different strategies. Red points represent negative labeled data, and green points represent positive labeled data. The boundary between the two classes is basically clear. Blue points ($F_{u}$) represent samples selected based on uncertainty strategy in an iteration, yellow points ($F_{d}$) are samples selected based on diversity strategy (according to (12)), and black points ($F_{u}$&$F_{d}$) represent selected samples that combine uncertainty and diversity.

We visually verify the proposed strategy to obtain an intuitive sense of effectiveness. Multi-dimensional scaling (MDS) is a commonly used solution for dimensionality reduction and visualization of high-dimensional data [55,56]. It can map distances between samples in high dimensions into distances in low dimensions and keep the relative length relation and order. We use the second-to-last fully connected layers in the classifier to perform dimensionality reduction on the data and then perform MDS on the dimensionality reduction result. The visualization result is shown in Figure 3.

Samples near the decision boundary often make the model make mistakes, so selecting and annotating the first will help speed up the improvement of model performance. This is also one of the motivations for us to introduce diversity. Many samples selected based on uncertainty strategy are falling at the center of the classes, not near the decision boundary of the sample, and this may result in a lot of not very effective annotation. The black points based on the two selection methods contain points near the decision boundary (where the diversity score is high) and also contain points away from the decision boundary (where the uncertainty score is high).

#### 3.2.1. Uncertainty

The uncertainty base strategy is widely used in active learning. It can be evaluated through the output $y^{\prime }$ of classifier M. Bayesian active learning (BALD) [57] was proposed by researchers. It follows (9):

(9) $$unc(x,M)=H\left[y^{\prime }|x,D^{L}\right]-E_{\theta \sim p\left(\theta |D^{L}\right)}H\left[y^{\prime }|x,\theta \right]$$

where unc(x, M) means the uncertainty of model M to sample x, and H[⋅] is Shannon entropy. The Monte Carlo (MC) dropout [58] method measures model uncertainty by obtaining the output of multiple rounds of predictions. The expression is as follows (10):

(10) $$\mathrm{unc}(x,M)\approx -\sum_{c}\left(\frac{1}{T}\sum_{t}{\hat{p}}_{c}^{t}\right)\log\left(\frac{1}{T}\sum_{t}{\hat{p}}_{c}^{t}\right)+\frac{1}{T}\sum_{c,t}{\hat{p}}_{c}^{t}\log\left({\hat{p}}_{c}^{t}\right)$$

where T is the number of dropout iterations, and ${\hat{p}}_{c}^{t}$ represents the probability that the sample is the c−th class in the t−th round of prediction.

#### 3.2.2. Diversity

A selection strategy based on BALD is not quite sufficient and may overfit on the selected samples [59,60]. Therefore, a diversity strategy is introduced to further accelerate performance improvement. As shown in Figure 3, the classifier is often easy to distinguish samples at the center of the category and prone to making mistakes for points in the intersection area or disagreement area. For example, in handwritten digit recognition, the number one and the number seven are similar and may have more large overlapping areas in high dimension manifold [61]. Selecting samples located in the disagreement area will help to acquire clearer decision boundaries, thus obtaining a greater performance improvement.

We believe that samples at the boundary of the category cluster tend to confuse the classifier, so we formulated a diversity score to quantify this property, and its expression follows (11):

(11) $${\mathrm{div}}_{i}=\sum_{k}\mathrm{softmax}\left(Z\left(d_{i,c}\right)\right)\log\left(\mathrm{softmax}\left(Z\left(d_{i,c}\right)\right)\right)$$

where $div_{i}$ represents the diversity score of sample i, Z(⋅) represents the Z-score operation, softmax(⋅) represents the softmax operation, and $d_{i,c}$ represents the distance from sample i to category c. k is the truncation coefficient. After sorting $d_{i,c}$ in ascending order, the first k elements are intercepted and used to calculate $div_{i}$. $d_{i,c}$ is measured by (12):

(12) $$d_{i,c}={||\frac{1}{\left|D_{c}\right|}\sum_{j\in D_{c}}z_{j}^{\prime }-z_{i}^{\prime }||}_{2}$$

where $d_{i,c}$ represents the distance from sample i to category c (we use the centroid of all samples as the category center), ${z^{\prime }}_{i}$ is the latent vector of sample i, $D_{c}$ is the marked sample set of class c, and $\left|D_{c}\right|$ is the sample size. The larger the $div_{i}$, the more balanced the distance of the sample to all class centroids and the more likely the sample is in the intersection area, through comprehensive consideration of the uncertainty and diversity of the sample, that is, according to (13):

(13) $$score=Scl\left(unc\left(x_{i},\;M\right)\right)+\left(1-\theta \right)Scl\left(div_{i}\right)$$

where score represents the comprehensive score of the sample, Scl(⋅) is the min–max scaling operation, and θ is the trade-off coefficient to weigh the effects of the two.

The entire model can be summarized as Algorithm 1.
**Algorithm 1.** Procedure of the proposed framework.
 **Require**: labeled dataset $D^{L}$, unlabeled dataset $D^{U}$
 **Ensure**: classifier M1: Construct and initialize class classifier M, encoder E, generator G, real/fake classifier D
2: Train M, E, D, G with $D^{L}$
3: **while** not satisfy condition **do**4:  randomly sample $x_{batch}$ from $D^{U}$
5:  calculate $score_{i}$ ∀ i ∈ $x_{batch}$ according to (13)6:  select n sample with the highest score7:  $D_{inc}\leftarrow \emptyset$
8:  **for** $x^{*}$ in $x_{re}$ **do**9:   query the label of $x^{*}$ from Oracle and get $y^{*}$ $x_{re}^{*}\leftarrow G\left(E\left(x^{*}\right)\right)$
10:   $x_{re}^{*}\leftarrow G\left(E\left(x^{*}\right)\right)$
11:   
$D_{inc}\leftarrow \;D_{inc}\cup \left\{\left(x_{re}^{*},y^{*}\right),\left(\;\;x^{*},\;\;y^{*}\right)\right\}$
12:   
$D^{L}\leftarrow D^{L}\cup D_{inc}$
13:  **end for**
14:  retrain M,E,D,C with $D^{L}$ by (2)–(8)15: return M


Assuming that the time for a round of forwarding propagation on *b* samples is *t_f_*, and the time for backward propagation is *t_b_*, and in each iteration, we train classifier e epochs. Then, the time consumed to train a normal classifier using n training samples is about $\frac{n}{b}e\left(t_{f}+t_{b}\right)$.

For the proposed strategy, we need to train four models: encoder, decoder, discriminator, and classifier in each query iteration. Assuming that the number of parameters of the four models is similar, the training time is about $4\frac{n}{b}e\left(t_{f}+t_{b}\right)$. When the training is over in an iteration, we need to query the unlabeled samples. When using MC Dropout to calculate the uncertainty, assume that the dropout is performed T times, and each time q samples are selected from p samples for labeling, the time cost is $4\frac{n}{b}e\left(t_{f}+t_{b}\right)+\left(T+1\right)\frac{p}{b}t_{f}$, where the latter item indicates the time required in the query. The number of training samples n is $n_{0},n_{0}+q,n_{0}+2q,n_{0}+3q,\ldots .n_{0}+mq$ in each iteration, where m is the total number of iterations. The total training time required for the entire iteration process is $4\frac{\left(2n_{0}+mq\right)\left(m+1\right)}{b}e\left(t_{f}+t_{b}\right)+m\left(T+1\right)\frac{p}{b}t_{f}$. Compared to the time spent by directly training the classifier, the proposed strategy training will be more time consuming.

However, the training time cost will not be an obstacle in its actual application. The first reason is that human experts cannot annotate the selected samples instantly; therefore, the high training cost will not be the bottleneck in terms of time cost. The second reason is that it is more expensive to hire doctors than hiring GPUs. The increase in training costs is beneficial compared to the decrease in annotation costs it brings.

## 4. Experiments and Results

### 4.1. Datasets and Configuration

The data in this experiment are from the Monash University Centre for Artificial Intelligence Research. The dataset includes more than 1000 histopathology scan images. After we process the dataset, there are a total of 17,000 color images. Directly using raw datasets to train models can impose serious burdens and costs on equipment. We resize the image to 200 × 200 to train the model more efficiently. After removing invalid data, there are 6826 valid pathological images. We set the ratio of the training and test sets to 7:3. The experiment was performed 10 times of cross-validation. Among them, the positive samples numbered 3904 or about 57%. The negative samples were 2922, accounting for approximately 43%. There was no sample imbalance. The difference in the proportion of positive and negative samples is small. To fairly evaluate the performance of each model, all models are experimented in the same environment using the same dataset.

We use convolutional neural networks as classification models. In the experiments, the number of samples (n in Section 3.2.2) selected for each iteration is 100, and the total number of iterations is 100. We use precision, recall, and AUC as evaluation metrics for the method [62]. In addition, we comparatively analyze the following methods:

(1) Random selection (Random): the core idea of this strategy is that in each iteration, we randomly select samples to label.
(2) BALD uncertainty-based strategy (denoted as BALD): in each iteration, we use BALD uncertainty to select samples for labeling.
(3) Other strategies based on uncertainty, including entropy-based strategies (entropy) and confidence-based strategies (confidence) [18,63].
(4) Strategy based on GAN reconstruction and BALD uncertainty (denoted as GAN + BALD): simultaneously train GAN and classifier, use BALD to select samples, and then augment the training set with reconstructed and selected images.
(5) Strategy (our) based on the method proposed in this study: train the GAN and the classifier simultaneously, select samples using Equation (13), and then augment the training set with the reconstructed and selected images.
(6) AIFT: It is a model that integrates active learning and transfer learning together. It is achieved by integrating active learning into fine-tuned CNNs in a continuous manner [64].
(7) O-MedAL: It is a strategy that uses new labeled samples and a subset of previously labeled samples to increment the model and improve the MedAL model by introducing online learning methods [65].

### 4.2. Data Processing

**Figure 4:**
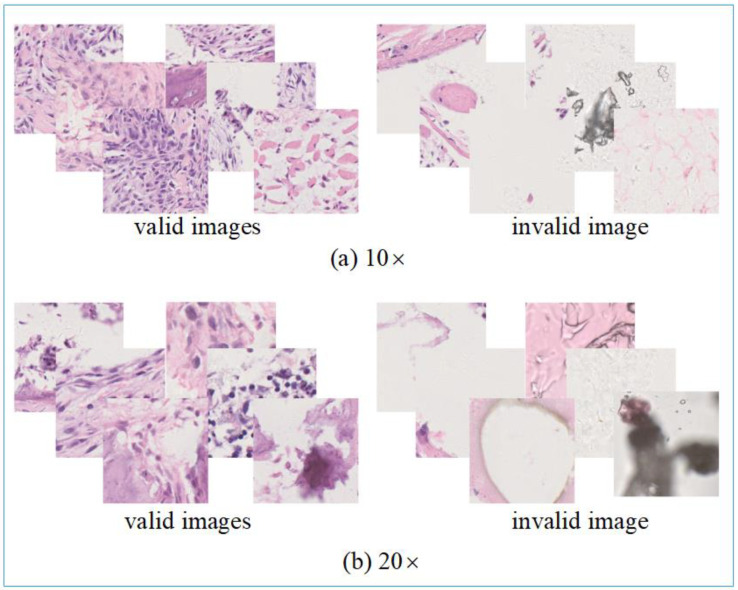
The example of data processing outcome. In the figure, the invalid images are the rejected samples, and the valid images are the sample data used for model training. Part (**a**) is the result of enlarging the original pathology image by 10×. Part (**b**) is the result of enlarging the original image by 20×.

In the osteosarcoma original pathology dataset, there are a total of 17,000 color images. There are many invalid images, as shown in Figure 4. Some color images do not include intact cells, or even only tissue fluid, or pathological sections are contaminated, resulting in unclear images, water spots, etc. To avoid ineffective resource waste, we screened the original osteosarcoma histopathology images. We removed invalid images from the dataset. After eliminating 10,174 invalid images, there are 6826 remaining valid color images. Of these, 4027 images were 10× magnification of the original slice, and 2799 were 20× magnification.

To prevent the model’s overfitting issue, we perform data enhancement after removing the invalid data. Firstly, each image is rotated by 90°, 180°, and 270°, and each image is flipped horizontally and vertically, at which time the amount of data is eight times that of the original dataset. Next, we select some images at random and add noise to them to reduce the overfitting phenomenon that occurs when the model learns high-frequency features. After data enhancement, the existing pathology samples reached 54,608.

### 4.3. Results

(1) Validation of hyperparameters

**Figure 5:**
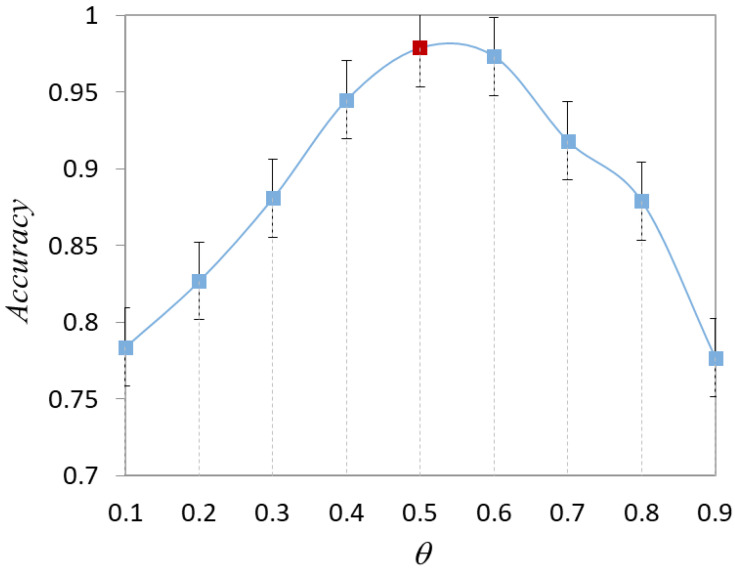
Effect of parameter θ on model accuracy.

The hyperparameter θ is introduced in the selection strategy combined with diversity. When there is no prior knowledge of the data distribution, the optimal value of θ is not known. To choose the best value, we conduct experiments to test the effect of different θ values on the model performance, as shown in Figure 5. As θ changes, the classification accuracy of the model changes dynamically. Throughout the training process, accuracy takes the lead in a continuous rise, reaches a critical point, and then continues to decline. With θ<0.5, the model focuses more on the diversity of the sample. With θ>0.5, the model focuses more on the uncertainty about the sample. The results demonstrate that the model has equal importance based on diversity and uncertainty.

**Figure 6:**
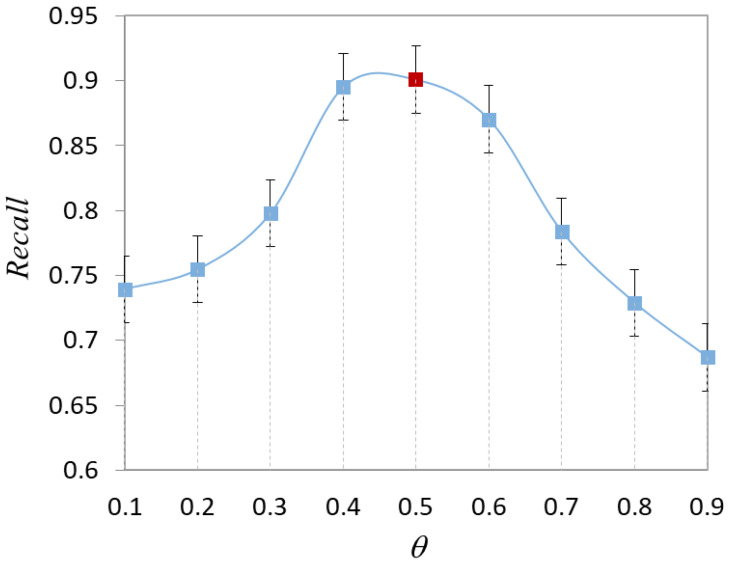
Effect of parameter θ on model recall.

The effect of the parameter θ on the recall of the model is shown in Figure 6. In the actual osteosarcoma pathology images, we are more concerned with model recall. This is mainly due to the more serious consequences of misclassifying abnormal images as normal images. The recall of the method has a significant change with the increase of θ. In particular, the recall rate reaches a critical value when the parameter θ increases from 0.3 to 0.7. The recall of the model is close to 0.9 when θ=0.4. It indicates that the model has a good classification effect at this time. With θ>0.5, the recall of the model gradually decreases.

After considering Figure 5 and Figure 6 together, we set θ=0.5 for the experiments. As the number of training rounds increases and the experimental data changes, the optimal θ may also be other values. The value of θ may change dynamically during the subsequent iterations.

(2) Analysis of the final results of model classification

Based on the above dataset and environment configuration, we iteratively trained the model. Table 2 shows the performance of Random, BALD, GAN + BALD, Entropy, Confidence, and our method in terms of AUC, Accuracy, and Recall. The AUC value of the Random strategy is the smallest, and the recall rate is also the lowest, only 0.77. Relatively speaking, the performance of the Entropy and Confidence methods has been greatly improved. AUC increased by 2.605% on average, and recall increased by 10.78% on average. Although the AUC value of GAN + BALD reaches 0.99 and the accuracy reaches 0.98, its recall rate is low. Compared to the first five methods, the AIFT model has little difference in each metric. Although it uses active learning to fine-tune the CNN, it finally achieves an AUC value of 0.9807, an accuracy of 0.9832, and a recall of 0.8712. All metrics are lower than those of the GAN + BALD strategy. The AUC value of the O-MedAL model is also lower than that of the GAN + BALD strategy, but its recall and accuracy are better. In the classification of osteosarcoma histopathological images, this is disadvantageous for the classification of osteosarcoma pathological images. In hospital diagnosis, doctors and patients will pay more attention to the recall rate. According to the experimental results, the indicators of our proposed method are the best. Not only does it have a precision close to 0.99, but the recall also hits 0.90. In particular, its recall rate is about 17.5% higher than that of GAN + BALD.

(3) Results of comparing different scaled datasets

**Figure 7:**
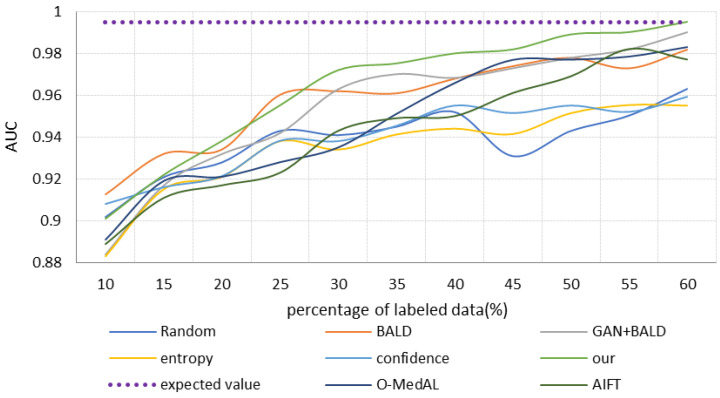
AUC with the increase of iterations.

**Figure 8:**
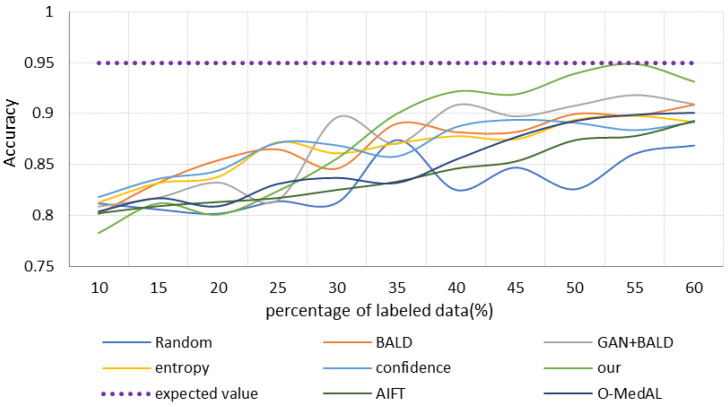
Accuracy with the increase of iterations.

One aspect of this research is to propose a high-precision osteosarcoma pathological image-assisted classification system. Another important purpose is to use fewer labeled samples to achieve similar accuracy to all data training, demonstrating the low cost and effectiveness of the selection strategy. Therefore, we retrain each model in turn by selecting the same proportion of samples with labels. Specifically, we choose 10% of the total labeled samples as the initial training set and then keep adding new labeled images at a rate of 5% for experiments. To avoid imbalance in the dataset, each time we selected the positive and negative images of the patients separately according to the same ratio. The performance of different strategies is shown in Figure 6, Figure 7 and Figure 8. The different colored lines represent several different selection strategies. Here, we use “convergence” to mean using part of the samples to train the classification model until achieving the same performance of using all the sample classification models, such as obtaining the same accuracy, recall, or AUC, that is, the purple dotted line is the result of training the base model with all the data. We end the iterative process with this value.

The relationship between AUC and training samples is shown in Figure 7. With the increase of training samples, the AUC of all strategies increases gradually. The BALD strategy has the highest AUC value when the training sample is only 10% of the total dataset, followed by Confidence, our method, Random, O-MedAL, and AIFT models, in that order. The Entropy strategy and the GAN + BALD method have the worst performance. The performance of the BALD strategy is the best when the percentage of training samples is less than 25%. GAN + BALD and the method proposed in this study have the largest increase in AUC values. For the AIFT model and the O-MedAL model, the AUC values of both are still at a low level, despite their improvement. The performance of the Random strategy is very unstable when the training sample is greater than 25%, and the AUC value has a substantial decrease when the training sample increases from 40% to 45%. The AUC value of the AIFT model has a large increase, especially when the training sample is 55%, and the AUC reaches 0.98. The AUC value of our proposed method is always the highest, and the “convergence” value is reached with only 60% of the training data. This is mainly due to the combination of uncertainty and diversity in our proposed strategy. Furthermore, it can be clearly seen that throughout the iteration process, the BALD strategy is always better than simply randomly selecting samples for training.

Figure 8 is the relationship between the accuracy of different strategies and samples. The Random strategy achieves an accuracy of 0.88 when the labeled sample is 35%, but it has a large fluctuation in accuracy when selecting samples throughout the process. This indicates that the Random strategy is random and not suitable for practical tasks. The accuracy of the BALD and GAN + BALD models is also at an unstable level, but the accuracy of the GAN + BALD model is always at a higher value. In most cases, the accuracy of the GAN + BALD model outperforms most of the other methods. The accuracy of the AIFT model is always increasing its state, but its value is only higher than that of the Random strategy. The accuracy of the Entropy strategy, BALD strategy, and Confidence strategy is relatively high when the training sample is less than 35%. During the increase of the labeled training sample from 35% to 60%, the Entropy, Confidence, and BALD strategies maintain a slowly increasing trend and are in a steady state. This also implies that the small increase of the dataset does not improve the model performance significantly. Our method consistently maintains the highest accuracy after reaching 35% of the training samples. In particular, when the training samples reach 50, the accuracy is close to the best value of the initial training, 0.95. In addition, at this time, the accuracy of our method is 7.7% higher than the GAN + BALD algorithm. Furthermore, it is evident that our proposed strategy is the first to achieve the accuracy of the base model training. This shows that it requires only a small amount of labeled data to achieve the best performance of the model.

**Figure 9:**
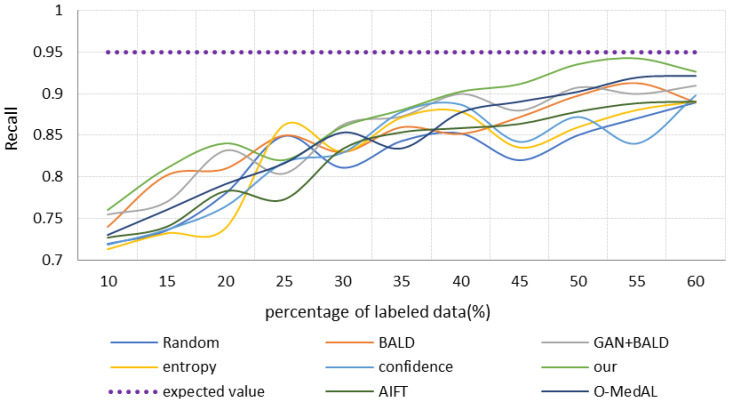
Recall with the increase of iterations.

The relationship between the recall rate of each method and the samples is shown in Figure 9. When the labeled data are only 10%, the recall of our method is the highest, followed by the GAN + BALD model. Both recall percentages are higher than 0.75. For the remaining models, their recall rates vary less and are between 0.7 and 0.75. Overall, the recall rate of each model gradually improves as the labeled sample increases. When the training samples reach 55%, the recall of our proposed method approaches 0.95 (“convergence” value). At this point, the O-MedAL model, BALD, and GAN + BALD strategies also have a recall of 0.9. The Confidence strategy has the lowest recall of less than 0.85. Then, the Random strategy, Entropy strategy, and AIFT model are in order. In comparison, the performance of the Entropy strategy, confidence strategy, and Random strategy is poor, and the recall rate is only about 0.85. Moreover, the recall rates of these three algorithms all fluctuate greatly with the increase of training data. Despite being only 35% of the training samples, the recall rate of the entropy and confidence strategies is above 0.85. However, they were always in an unstable state, suggesting they were less effective.

The strategy that takes into account uncertainty and diversity converges faster than the strategy that only relies on uncertainty. It is worth pointing out that in the early stages of iterative training, the quality of GAN reconstruction samples is not high. This is because the number of labeled samples is small, and the global information of the data distribution captured by GAN is less. Therefore, strategies combined with GAN perform worse than the pure uncertainty strategy. However, in the later stage, as the quality of GAN-reconstructed samples improves, strategies combined with GAN gradually gain the upper hand, especially our proposed method. It first reaches the “convergence” value and then maintains a stable and efficient state.

**Figure 10:**
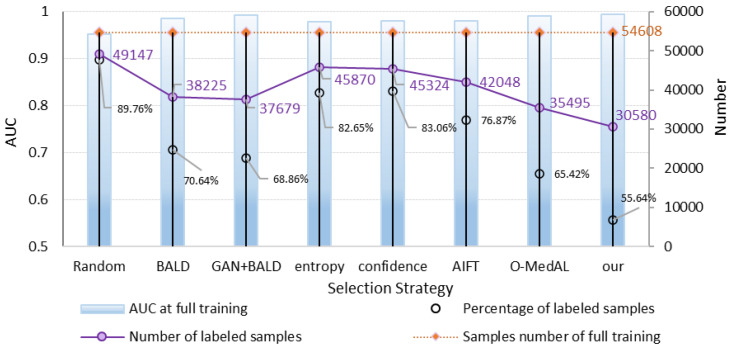
Performance and training sample number comparison of the full-training and proposed framework.

Figure 10 compares the performance and the number of training samples for the fully trained and proposed frameworks. When different methods achieve and fully train the same performance, there is a large difference in the amount of labeled data used. As can be seen from the results presented in Figure 8, our method requires only 55.64% of the labeled data for training when it achieves the same performance as training with all labels. This is very valuable when assisting in the analysis of pathological images of osteosarcoma in actual hospitals. The reduction in the need for labeled data means a significant reduction in the cost of annotation.

(4) Analysis of the results of physician verification

From the prediction results of each stage of our model, the doctors randomly selected 100 images for validation to measure the model’s accuracy from a medical standpoint. As shown in Table 3, the predicted values are the prediction results of the model when trained with different proportions of labeled samples. The actual values are the results of the randomly selected samples evaluated by a physician after the examination. When using a smaller number of labeled samples (10% of the labeled data), the system predicts less accurate results. For example, the model output determined 45 images to be positive, but after the doctor’s examination, only 21 images were actually positive. The model predicts 55 images as negative, but only 25 images are actually negative. As more labeled training samples are added, the model’s performance gradually becomes better. When the trained labeled data reached 50%, the prediction accuracy improved significantly to 0.74. After all the data had been fully trained, the model had an accuracy and recall of 0.89 and 0.88, respectively. This basically agrees with the findings of the analysis mentioned above. It shows that our proposed method has good performance. In a practical setting, it can be used as an auxiliary basis for doctors’ clinical diagnosis to improve the accuracy and efficiency of diagnosis and reduce the workload of doctors. Especially in the analysis of medical images, it only needs less annotation cost to achieve high performance, which effectively reduces the cost of building an intelligent medical assistance system.

### 4.4. Discussion

The performance of the model classification is not guaranteed when using the Random selection strategy because the chosen training samples have a high degree of randomness. Although the model’s overall performance improves as the number of training samples rises, there is little correlation between the performance improvement and the selection method. This is primarily because growing the training sample size improves the algorithm’s performance. Uncertainty-based strategies [18,60] (including Entropy and Confidence) are an effective improvement on the Random strategy. Such methods not only end up with higher classification accuracy, but they are able to achieve better accuracy with relatively less labeled data. When model iterations increase, a single BALD strategy may cause overfitting of local samples, which limits the performance of BALD strategies. GAN learns knowledge from the overall data distribution and adds it to the reconstructed samples to increase the richness of the data. Therefore, it can greatly improve the classification performance of the model. However, in the early stage of model training, when the number of labeled samples is small, the performance of BALD is better than that of the GAN + BALD model. This is mainly because GAN acquires less global information of the data, and the quality of the reconstructed samples is not high. As the training increases and the number of labeled samples increases, the quality of GAN-generated samples improves, and the performance of the GAN + BALD strategy has more substantial improvement.

Both the AIFT model [64] and the O-MedAL model [65] are based on active learning, but both have improved performance compared to strategies based purely on active learning or randomly selected labeled samples. The AIFT model is an approach that concentrates active learning and transfer learning into a single framework. Starting with a pre-trained CNN, AIFT looks for “valuable” annotated samples and progressively enhances the CNN by continuous fine-tuning. Because AIFT actively selects the most informative and representative samples, it also outperforms most experimental methods. The O-MedAL model samples unlabeled images by maximum uncertainty and distance from labeled samples. As the model improves the classification accuracy of the samples, the recognition of unlabeled samples is also increasing. It uses relatively few samples and achieves the same performance as when fully trained.

The results show that our OHIcsA method has the best performance. Not only does it have the highest classification accuracy when fully trained, but it requires only 60% of the training samples to achieve similar performance as when trained with full samples with labels. It significantly reduces the cost of building such a medical system. This is mainly since our approach combines active learning and generative models. The active-learning strategy is designed to combine sample class diversity and uncertainty to select “more valuable” samples, and the GAN model with strong learning data distribution capability generates pseudo-samples to extend the original dataset. This approach results in a faster performance improvement of the model. However, since our selection strategy focuses on selecting “valuable” or “informative” images, in the early stage of model training, there may be anomalous cases of selected images, and the quality of CACEGAN reconstruction samples is low. Therefore, the performance of the model is not outstanding at this stage. With the iteration of the model and the increase of training data, the performance of our method improves more substantially. In general, our method is the first to reach the “convergence” value, and thereafter it ensures a relatively stable and efficient level. It effectively overcomes the problem of low medical labeled data and achieves better classification performance with fewer labeled samples.

## 5. Conclusions

The study proposes an active-learning-based method for histopathological images of osteosarcoma for assisted classification. The system exploits the powerful fitting ability of deep convolutional networks to improve classification performance and uses a combination of active learning and generative adversarial networks to obtain faster performance gains. On the one hand, GAN generates more realistic pseudo-samples that augment the original dataset. On the other hand, the strategy of combining diversity and proactivity is used to select samples with a large amount of information. The classification results can be used as an aid for physicians to identify pathological images of osteosarcoma, reducing the workload and misdiagnosis rate in osteosarcoma diagnosis. More importantly, the method requires only a small amount of labeled data to achieve similar performance when trained on all labeled data. It is designed mainly to reduce the cost of labeled data and building the system.

In the future, as computing power grows and deep-learning methods advance, we will pay more attention to how well model training works in its initial stages. Our research will concentrate on finding solutions to the issues of low sample quality produced by GAN and anomalies of the images chosen by the diversity strategy. Additionally, enhancing model training effectiveness will be a key concern of our strategy.

## Figures and Tables

**Table 1:** Partial abbreviation vocabulary and basic symbols interpretation table.

Symbol	Meaning
x	The true sample
$D^{L}$	Labeled data
$D^{U}$	Unlabeled data
$x_{re}$	Reconstructed samples
$x_{p}$	Pseudo-samples
KL(⋅)	Kullback–Leibler divergence operator
$L_{D}$	The loss of discriminator D
$L_{C}$	The loss of classifier M
$L_{KL}$	The difference between distribution N(0,1) and posterior probability distribution of *z*
$L_{G}$ $,\;L_{GD}$ $,\;L_{GC}$	The loss of generator G
unc(x, M)	The uncertainty of model M to sample x
H[⋅]	Shannon entropy
T	The number of dropout iterations
${\hat{p}}_{c}^{t}$	The probability that the sample is the c−th class in the t−th round of prediction
$div_{i}$	The diversity score of the sample i
$d_{i,c}$	The distance from sample i to category c
θ	Trade-off factors for diversity and uncertainty

**Table 2:** Performance of different strategies at the end of the iterative process.

Selection Strategy	AUC	Accuracy	Recall
Random	0.9546	0.9830	0.7729
BALD	0.9856	0.9829	0.7922
GAN + BALD	0.9931	0.9849	0.8743
Entropy	0.9786	0.9789	0.8598
Confidence	0.9804	0.9804	0.8526
AIFT	0.9807	0.9832	0.8712
O-MedAL	0.9918	0.9854	0.8894
Our	0.9950	0.9890	0.9080

**Table 3:** Analysis of model prediction results and physician examination results.

Percentage of Labeled Samples		True	Positive	Negative
	Predicted			
10%	Positive		21	24
	Negative		30	25
50%	Positive		41	15
	Negative		11	33
100%	Positive		46	5
	Negative		6	43

## Data Availability

Data used to support the findings of this study are currently under embargo while the research findings are commercialized. Requests for data, 12 months after the publication of this article, will be considered by the corresponding author.
